# Supplementary material for: Baseline Characterization of the Gut Microbiota of Field and Colony Populations of Phlebotomus tobbi and Preliminary Assessment of the Anti-Leishmanial Activity of Cultivable Bacteria
Source: Pathogens. 2026 Jun 23;15(7):658. doi: 10.3390/pathogens15070658 (PMC13416292; doi:10.3390/pathogens15070658)
Supplement: Supplementary file 1 [file pathogens-15-00658-s001.zip › pathogens-4357168-File S1/Taxa2ASV_Decomposer/Genus/g__Serratia/abun.plot.top12.pdf]

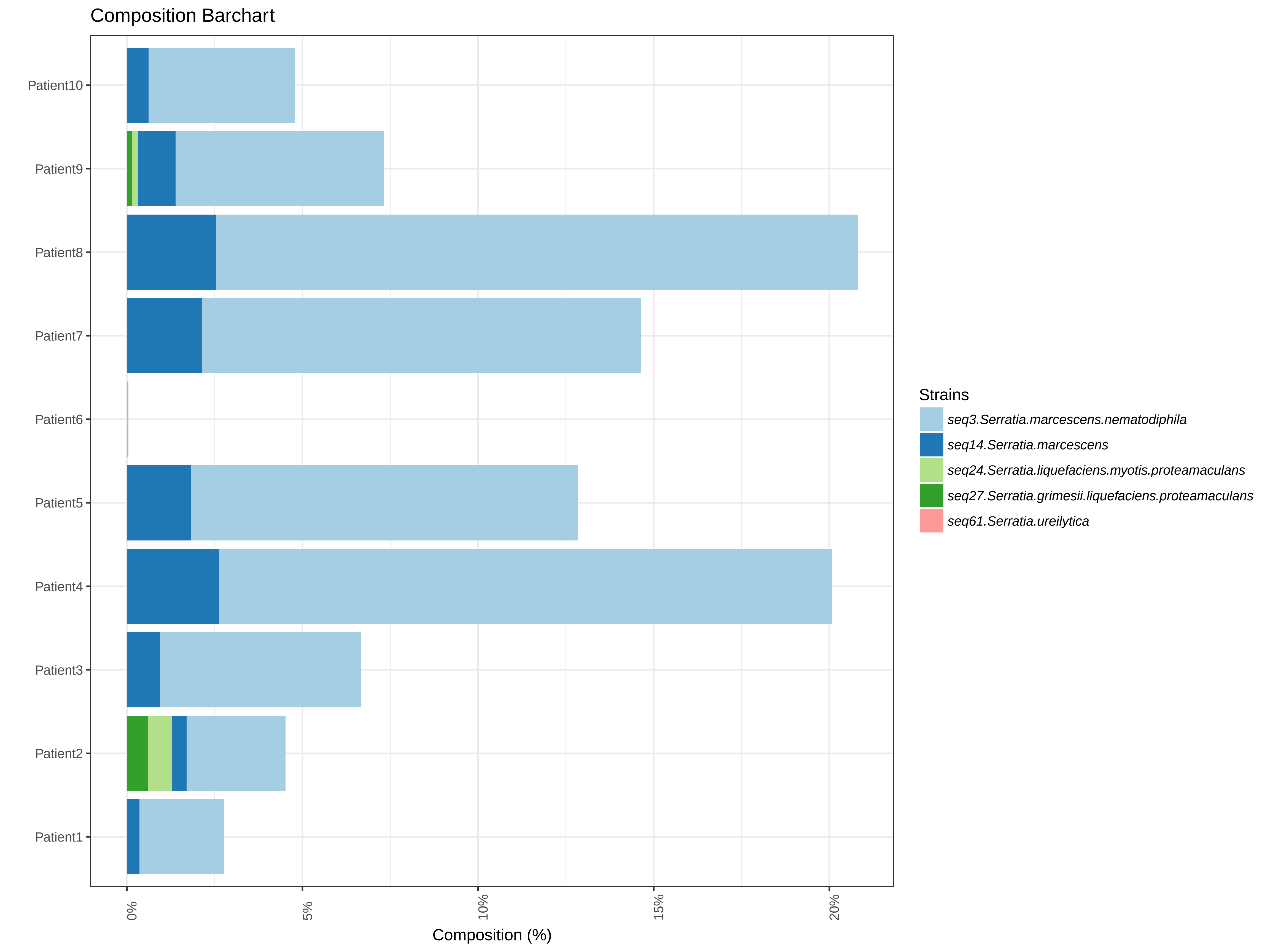

Composition Barchart
Patient10
Patient9
Patient8
Patient7
Patient6
Patient5
Patient4
Patient3
Patient2
Patient1
0%
5%
10%
15%
20%
Composition (%)
Strains
seq3.Serratia.marcescens.nematodiphila
seq14.Serratia.marcescens
seq24.Serratia.liquefaciens.myotis.proteamaculans
seq27.Serratia.grimesii.liquefaciens.proteamaculans
seq61.Serratia.ureilytica
